# A Woman With a Distended Abdomen

**DOI:** 10.1016/j.acepjo.2026.100509

**Published:** 2026-09-18

**Authors:** Ariel Vinson, Stevley Koshy, Derrick Huang

**Affiliations:** Department of Emergency Medicine, University of Houston/HCA Emergency Medicine Residency Program at HCA Houston Clear Lake Hospital, Houston, Texas, USA

## 1 Patient Presentation

A woman with a distended abdomen.

## 2 Case Presentation

**Figure 1:**
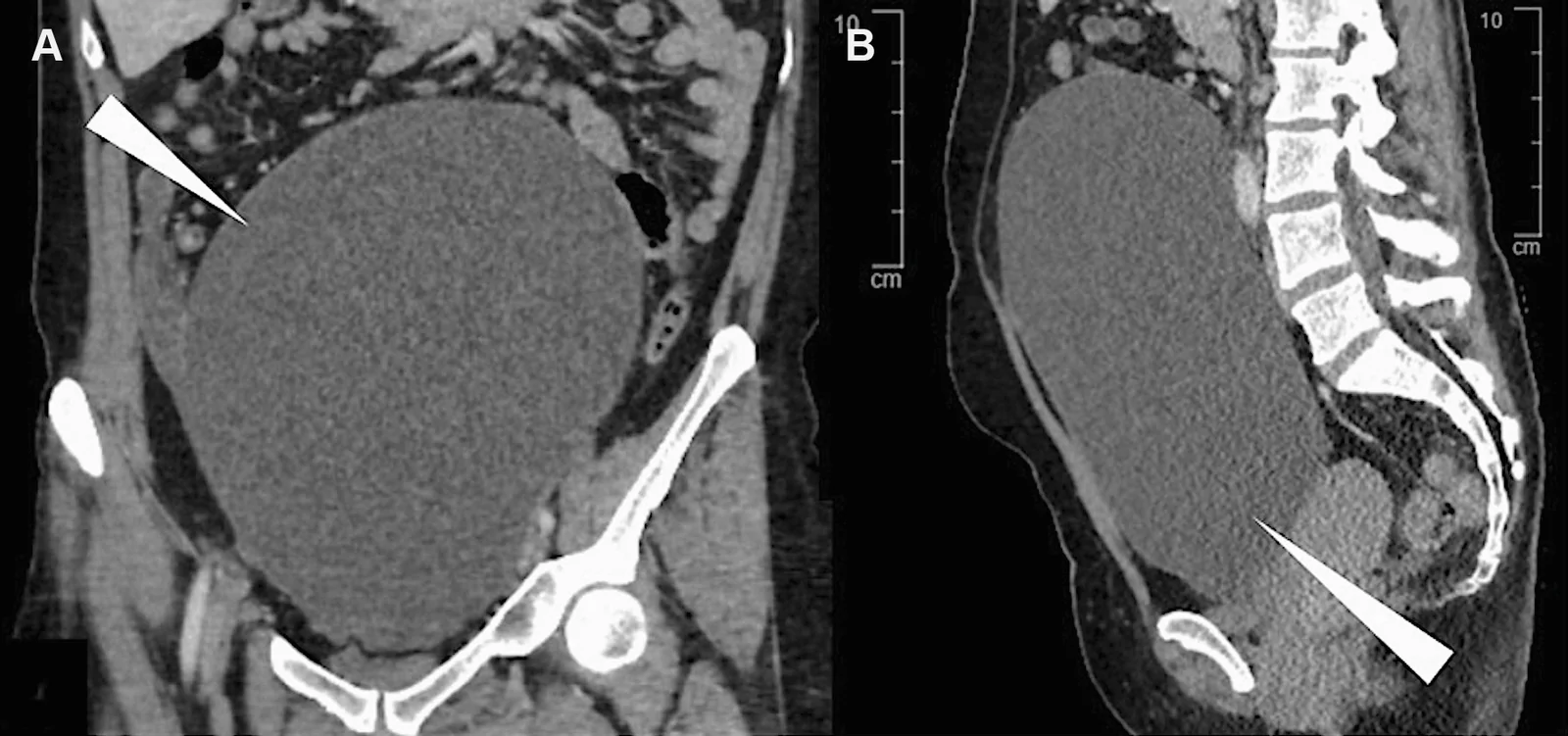
CT abdomen and pelvis with contrast in (A) coronal and (B) sagittal views, showing distended suprapubic mass (white arrowheads). CT, computed tomography.

**Figure 2:**
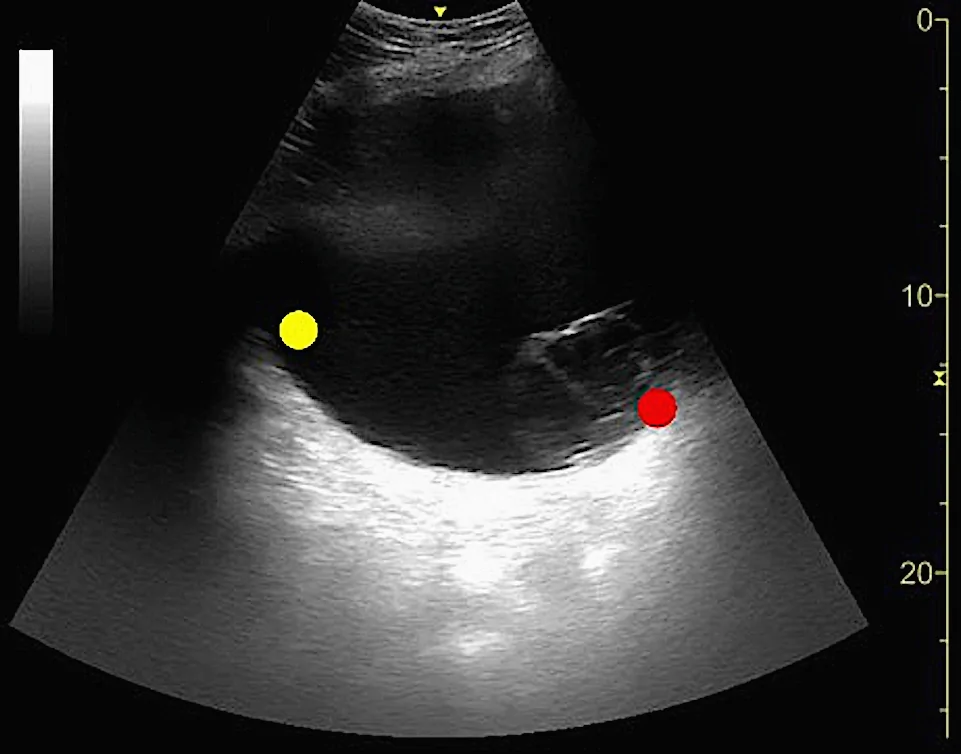
Bedside point-of-care ultrasound image with a low-frequency curvilinear probe in transverse orientation, visualizing an anechoic, fluid-filled structure (yellow circle) with an internal cystic mass with septations (red circle).

**Figure 3:**
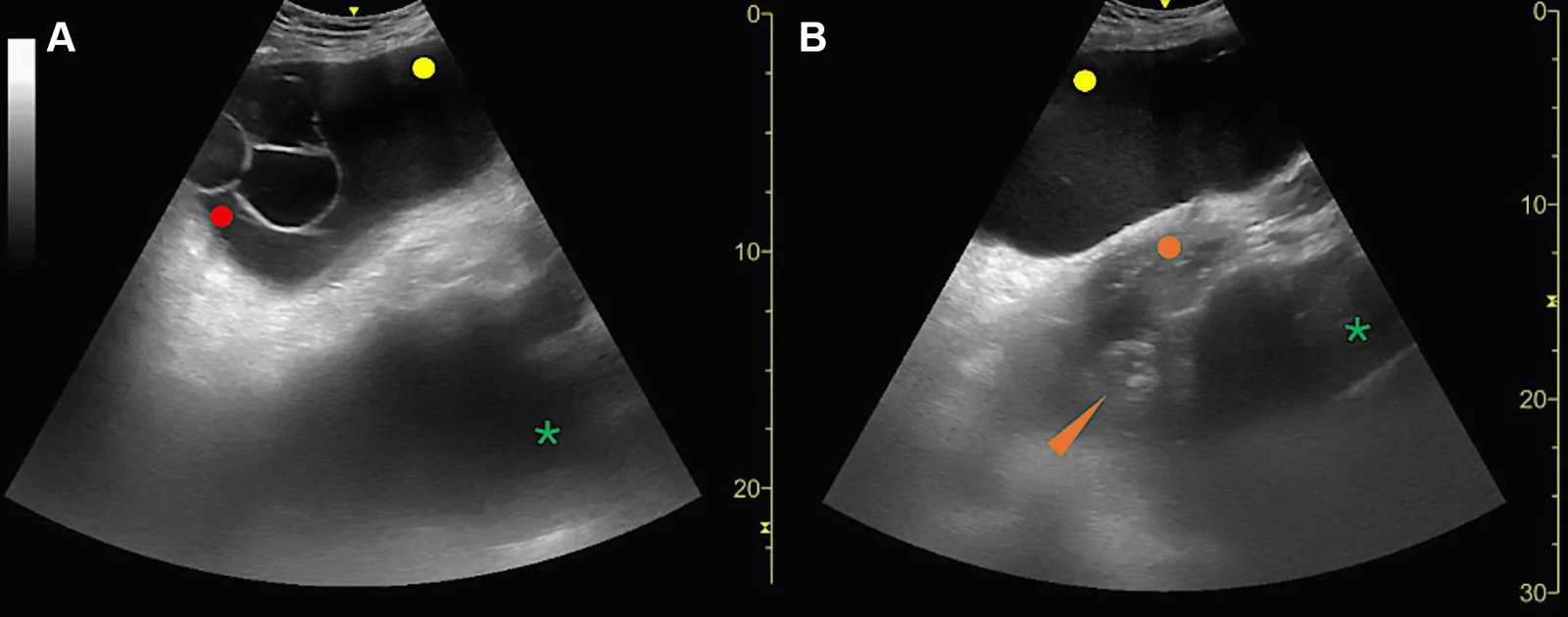
Bedside point-of-care ultrasound image with a low-frequency curvilinear probe in sagittal orientation, visualizing (A) anechoic, fluid-filled structure (yellow circles) with an internal cystic mass with septations (red circle) and (B) a Foley catheter (orange arrowhead) in a collapsed bladder (orange circle). Bowel is visualized deep to the fluid-filled structure and bladder (green asterisks).

A 28-year-old female presented with abdominal distention and pain beginning several hours before arrival. On exam, she had a tender, distended abdomen. Bladder scanning estimated a bladder volume of >999 mL, whereas computed tomography (CT), demonstrated severe bladder distention with a volume of 3.2 L (Fig 1). Foley catheterization was attempted with minimal output, prompting use of point-of-care ultrasound (POCUS), which revealed a large anechoic cystic mass with the Foley in a collapsed bladder (Fig 2,3). The patient was subsequently treated with a laparoscopic bilateral salpingectomy with left oophorectomy. Surgical pathology revealed a left ovarian mucinous cystadenoma of 18.8 cm.

## 3 Diagnosis: Mucinous Cystadenoma of The Left Ovary

Ovarian tumors are categorized as epithelial, stromal, or germ cell.^1^ Mucinous cystadenomas make up 15-20% of all epithelial tumors, and 80% are benign.^2^ These cysts present with progressive abdominal distension and pain, vaginal bleeding, and symptoms of organ compression, such as constipation and early satiety, commonly mimicking pathologic fluid-filled collections, such as ascites and bladder distention.^3^^,^^4^ The initial CT report described the pelvic fluid collection as a distended bladder, whereas POCUS differentiated the bladder from a cystic mass while also visualizing the collapsed bladder (Fig 2,3).

Although CT is superior to ultrasound for suspected genitourinary or gastrointestinal etiologies and to evaluate for metastasis if suspected on ultrasound, clinical assessment, and serum markers, CT has poor pelvic organ soft-tissue contrast, rendering this modality situationally suboptimal compared with ultrasound.^5^, ^6^, ^7^ POCUS has significant advantages over the bladder scanner, as it can differentiate free fluid, bowel obstruction, bladder distention and rupture, and cystic masses with septation, as well as assess for hydronephrosis, blood clots, and Foley dysfunction.^4^^,^^8^, ^9^, ^10^, ^11^, ^12^

## Ethics declaration

Written informed consent to take part in the study and to publish the article has been obtained from all participants or their legal representatives. The privacy rights of participants have been observed.

This study was performed in compliance with relevant laws, regulatory frameworks and guidelines where the research took place. Ethics committee approval was not required under relevant laws and institutional guidelines. Institutional Review Board (IRB) approval was not required for this manuscript, as it describes a retrospective case report of a single patient, which falls outside the definition of human subjects research under institutional guidelines.

This research follows the CARE guidelines and the CARE checklist.

## Funding and Support

By *JACEP Open* policy, all authors are required to disclose any and all commercial, financial, and other relationships in any way related to the subject of this article as per ICMJE conflict of interest guidelines (see www.icmje.org). This research was supported (in whole or in part) by HCA Healthcare and/or an HCA Healthcare affiliated entity.

## Conflict of Interest

None of the authors have any conflicts of interest to disclose.
